# Innate Lymphoid Cells as Regulators of Epithelial Integrity: Therapeutic Implications for Inflammatory Bowel Diseases

**DOI:** 10.3389/fmed.2021.656745

**Published:** 2021-03-30

**Authors:** Anja Schulz-Kuhnt, Markus F. Neurath, Stefan Wirtz, Imke Atreya

**Affiliations:** ^1^Department of Medicine 1, University Hospital of Erlangen, Erlangen, Germany; ^2^Deutsches Zentrum Immuntherapie, Erlangen, Germany

**Keywords:** innate lymphoid cells, intestinal epithelium, inflammatory bowel diseases, cytokines, ILC plasticity, colitis

## Abstract

The occurrence of epithelial defects in the gut relevantly contributes to the pathogenesis of inflammatory bowel diseases (IBD), whereby the impairment of intestinal epithelial barrier integrity seems to represent a primary trigger as well as a disease amplifying consequence of the chronic inflammatory process. Besides epithelial cell intrinsic factors, accumulated and overwhelmingly activated immune cells and their secretome have been identified as critical modulators of the pathologically altered intestinal epithelial cell (IEC) function in IBD. In this context, over the last 10 years increasing levels of attention have been paid to the group of innate lymphoid cells (ILCs). This is in particular due to a preferential location of these rather newly described innate immune cells in close proximity to mucosal barriers, their profound capacity to secrete effector cytokines and their numerical and functional alteration under chronic inflammatory conditions. Aiming on a comprehensive and updated summary of our current understanding of the bidirectional mucosal crosstalk between ILCs and IECs, this review article will in particular focus on the potential capacity of gut infiltrating type-1, type-2, and type-3 helper ILCs (ILC1s, ILC2s, and ILC3s, respectively) to impact on the survival, differentiation, and barrier function of IECs. Based on data acquired in IBD patients or in experimental models of colitis, we will discuss whether the different ILC subgroups could serve as potential therapeutic targets for maintenance of epithelial integrity and/or mucosal healing in IBD.

## Introduction

Worldwide, approximately seven million patients are diagnosed with inflammatory bowel diseases (IBD) (1), a chronically remitting inflammatory disease of the gastrointestinal tract. Both entities of IBD, Crohn's disease (CD) and ulcerative colitis (UC), commonly lead to severe intestinal symptoms like chronic diarrhea, abdominal pain, rectal bleeding, and anemia, thus significantly limiting the physical fitness, work ability, and overall quality of life of the affected and often young patients (2–4). Due to a complex and multifactorial pathogenesis, which is crucially influenced by genetic, environmental, microflora-related, and immunological components, there still exists no causal therapy for IBD (5, 6). Instead, established treatment strategies focus on the control of clinical symptoms, mainly by either inhibiting the overwhelming accumulation and activation of intestinal immune cells or by promoting mucosal healing (7, 8). During the last two decades, the spectrum of available therapeutic regimens underwent a significant shift from a predominance of rather unspecific immunosuppressive drugs, like glucocorticoids, thiopurines, and methotrexate, toward an increasing and often preferential use of biological agents, like anti-TNF antibodies, the anti-IL-12/IL-23 antibody ustekinumab or anti-adhesion strategies, which in principle allow to specifically interfere with disease-driving signaling cascades (8). However, still approximately 40% of IBD patients do not show a satisfactory primary response even to these optimized therapeutic regimens or develop a secondary loss of response (5, 9), emphasizing the urgent need to further fine-tune the definition of therapeutic target structures, and/or to better take into account the full spectrum of interacting cellular players involved in the pathogenesis of IBD. In this context, it will be of particular importance to better elucidate the complex crosstalk between intestinal epithelial cells (IECs) and subepithelial innate immune cells, which together form an early and tightly regulated line of host defense against invading luminal pathogens. Consequently, achieving improved insights into the capacity of lamina propria innate immune cells to interfere with the maintenance of epithelial integrity or the resolution of epithelial defects might subsequently pave the way for therapeutic approaches that pursue a two-pronged strategy: Epithelial restoration and suppression of overwhelming immune activation.

Within the mucosal immune cell compartment, the group of innate lymphoid cells (ILCs) can be characterized by their typical localization in direct spatial proximity to the epithelial surface (10, 11) and by their exceptional capability to initiate an early and rapid response to invading pathogens and epithelial damage (12–14). Although resembling T cells morphologically and in several functional aspects, ILCs are classic representatives of the innate immune system and can thus be distinguished by the lack of rearranged antigen-specific receptor expression and their ability to achieve a full activation status independent from the antigen-presentation and -recognition machinery. This predisposes ILCs for promoting and regulating early defense mechanisms (13). In analogy to T helper (Th) cells and mainly based on their cytokine and transcription factor profile, mature helper ILCs can be categorized into type-1 (ILC1s), type-2 (ILC2s), and type-3 ILCs (ILC3s) (15). Accordingly, ILC1s are associated with type-1 immune responses classically directed against intracellular pathogens and tumor cells, functionally depend on the transcription factor T-bet and preferentially secrete the pro-inflammatory cytokines IFN-γ and TNF-α (16, 17). Besides helper ILC1s, NK cells represent another ILC1 subset, which is characterized by a potent cytotoxic effector function (16) and whose impact on the maintenance of mucosal homoeostasis has been reviewed elsewhere (18). As an important cellular source of the effector cytokines IL-4, IL-5, IL-9, and IL-13 and, hereby, as an integral cellular component of mucosal type-2 immune responses, ILC2s are crucially involved in the immunological control of parasitic worm infections and in allergic diseases. Like in Th2 cells, GATA-3 and RORα represent important signature transcription factors in ILC2s (19–21). Finally, ILC3s depend on the transcription factor RORγt and can be subdivided into lymphoid tissue inducer (LTi) cells, which play a significant role during lymphoid organogenesis, as well as NCR^+^ and NCR^−^ ILC3s as relevant producers of IL-17A, IL-22, and GM-CSF at mucosal sites, appearing accordingly in many functional aspects like innate counterparts of Th17 cells (22, 23). An important functional attribute, which is common to all local helper ILC pools in mucosal organs, is their early activation in response to pathological tissue damage and their subsequent capacity to support, amplify, and modulate local adaptive immune responses (24, 25). While this sequential cascade of primary or externally triggered epithelial damage, subsequent ILC activation via epithelial cell-released factors and, finally, the ILC-mediated modulation of adaptive immune responses has drawn a lot of scientific attention during the last decade (15, 24, 26, 27), the opposite direction, meaning the ILC-to-epithelium communication, often seemed to fade a bit into the background. However, several studies described a significant influence of the ILC compartment on tissue regeneration, resolution of inflammatory tissue damage, and mucosal barrier integrity (28–32), although several details of the underlying mechanisms and the particular contribution of the different types of epithelial cells still remain incompletely understood. Due to the implicated high significance of the bidirectional crosstalk between locally accumulating ILCs and epithelial cells for maintenance of mucosal homeostasis in the gastrointestinal tract and its potential therapeutic targetability in IBD, we will here summarize the insights in the capacity of helper ILCs to influence the fate of different epithelial cell types in the gut under physiological and inflammatory conditions and discuss potentially resulting therapeutic perspectives.

## Intestinal ILCs in Healthy and Chronic Inflammatory Conditions

**Figure 1:**
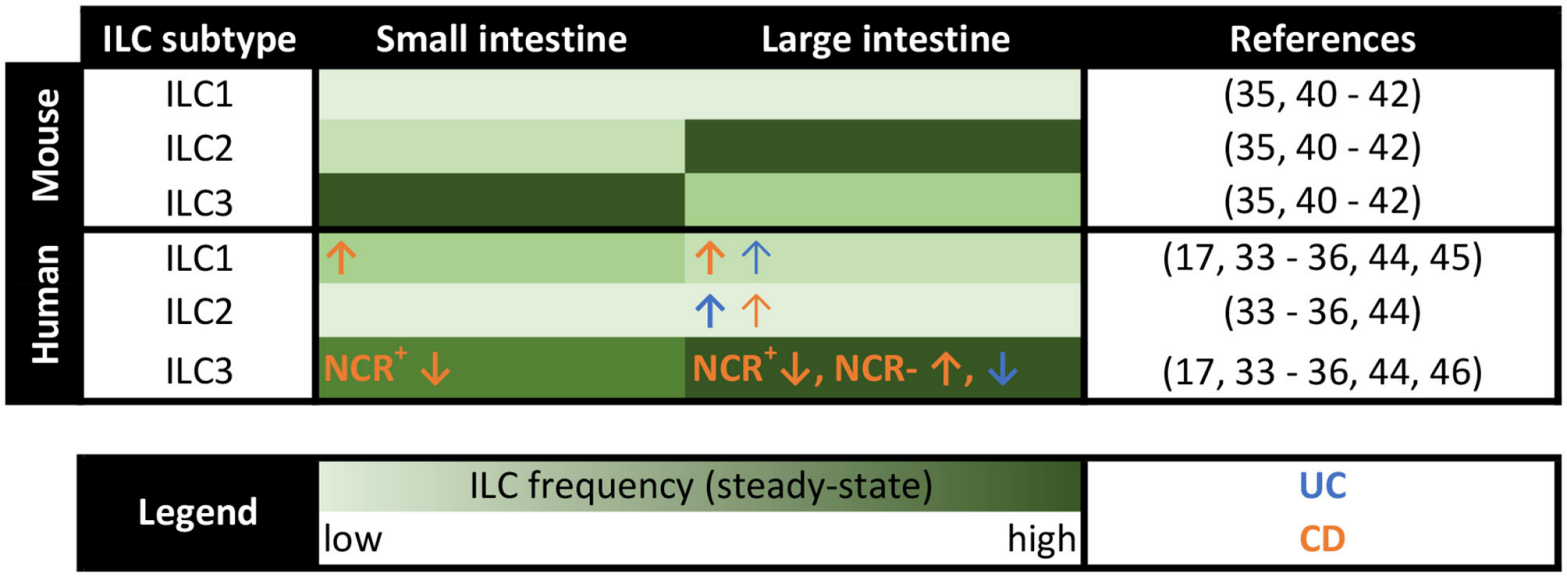
Distribution of murine and human ILCs along the intestine in steady-state and IBD patients. Heatmap summarizing the distribution of the classical helper ILC subsets in the lamina propria of the small and large intestine in both mice and humans. The frequency of ILCs is color-coded with the light green color indicating low frequencies and the dark green color representing high ILC percentages. Changes in local ILC frequencies in patients with ulcerative colitis (UC) and Crohn's disease (CD) are indicated by blue and orange arrows, respectively.

The distinct distribution of the three classical helper ILC subtypes observed at different anatomical sites upon homeostasis implies a specific function of each local subset in non-diseased tissues (Figure 1). In the human gastrointestinal tract NKp44^+^ ILC3s represent the main helper ILC population in the caecum, ileum, and colon, while ILC1s predominantly populate the upper gastrointestinal tract, including esophagus, stomach, and duodenum (33, 34). In contrast, ILC2s constitute only a small ILC population throughout the whole healthy intestine (33–36). However, when differentiating between lamina propria and intraepithelial ILCs more precisely, a distinct ILC2 population could be observed in the intraepithelial compartment of the colon together with a large ILC1 pool and only a minor fraction of ILC3s (35), suggesting a distinct function of each helper ILC subset in the gut under homeostatic conditions. In addition to the three classical helper ILC subsets, regulatory ILCs (ILCregs), which resemble regulatory T cells in several key features, were suggested to exist in both human and murine intestines. ILCregs were initially defined by their constitutive expression of IL-10 and were found to primarily accumulate in the lamina propria of the small intestine (37). Their existence, however, is still a matter of controversial discussion, given the inability of Bando and colleagues to detect ILCregs in the murine gut based on the expression of IL-10 in their study. Furthermore, they could not identify a helper ILC subset distinct from ILC1s, ILC2s, and ILC3s using mice from different breeding facilities in both steady-state and under inflammatory conditions (38). Instead, the authors described ILC2s as inducible source of intestinal IL-10 production (38), which is in line with the findings from other groups (39), raising the idea of IL-10-producing ILC2s rather than the existence of a distinct ILCreg subset.

Importantly, the murine intestine is populated by differently distributed ILC classes compared to human intestines (Figure 1). Unlike in humans, ILC2s make up a clear intestinal cell population in naive mice, which even outnumbers ILC3s or ILC1s in the lamina propria of the large intestine (35, 40). In the intraepithelial compartment and the small intestine, in contrast, murine ILC3s turned out as dominant ILC subtype (35, 41, 42). In total, murine ILCs were specifically enriched in the lamina propria of the large intestine (35). Keeping the species-specific differences in mind is important when assessing the translational relevance of results obtained in the murine organism. Additionally, the use of immunodeficient Rag1^−/−^ mice and, thus, the absence of functional adaptive immune cells in many murine *in vivo* ILC studies might bias the obtained results (13), emphasizing the need for confirmatory human studies. Nevertheless, the ability of highly controlled breeding, housing, and the availability of elegant genetic knockout mouse models, makes murine studies in the field of intestinal ILCs inevitable. The intensified consideration of humanized mouse models, in which the function of primary human ILCs can be analyzed under experimentally defined *in vivo* conditions, might even allow for better transferability of acquired data to the clinical context of human diseases (43).

Significant alterations in local ILC pools were observed in inflamed areas in IBD patients compared to unaffected control tissue (Figure 1), indicating a functional role of ILCs in chronic inflammation of the gut. While NKp44^+^ ILC3s constitute the dominant helper ILC population in the lower gastrointestinal tract in homeostasis (33, 34), their frequency was markedly reduced at sites of active inflammation in patients suffering from IBD, including both UC and CD (17, 34, 44). This ILC3 decrease further correlated with severe disease cases (34), highly suggesting a regulatory or protective function of ILC3s in intestinal inflammation. Contrary to NKp44^+^ ILC3s, the percentage of ILC1s, ILC2s, and NKp44^−^ ILC3 was found to be increased in IBD patients (34, 44–46). Especially in CD patients an enhanced percentage of intestinal ILC1s has been described in multiple studies (17, 34, 44) and was obviously associated with an advanced disease severity (34). Regarding the underlying mechanism for the accumulation of ILC1s in the inflamed intestine of CD patients, transdifferentiation of other ILC subtypes into ILC1s was suggested to take place in the IL-12-enriched microenvironment of the inflamed gut of CD patients (25). In *in vitro* experiments, ILC2s, ILC3s as well as c-Kit^+^NKp44^−^ immature ILCs were described to transdifferentiate into IFN-γ-secreting ILC1-like cells in the presence of IL-12 (17, 36, 47–50). And indeed, an increased local secretion of IL-12 was reported in CD patients (51, 52). Moreover, the biological relevance of this *in vitro* induced ILC3-to-ILC1 transition could be reinforced by an inverse link of ILC3 and ILC1 frequencies in the inflamed mucosa of CD patients (17, 34, 44) and the presence of an ILC subgroup harboring both ILC3 and ILC1 characteristics in human ileal LPMCs (53). Similarly, IL-13^+^IFN-γ^+^ ex-ILC2s were detected in the intestine of CD patients (48), hinting at ILC2-to-ILC1 transitions *in vivo*. In contrast to patients with CD, the intestinal tissue of UC patients was associated with an accumulation of NKp44^−^ ILC3s, which correlated with severe illness (34), making ILC1s and NKp44^−^ ILC3s specifically important in CD and UC, respectively. Although the scientific debate on the existence of ILCregs is still ongoing (37, 38), experimental models of innate colitis revealed an enhanced frequency of IL-10-producing ILCs, which the authors defined as ILCregs, upon intestinal inflammation, temporally following an increase of ILC3s and ILC1s. Accordingly, the transfer of ILCregs into *Rag1*^−/−^*Il10*^−/−^ mice resulted in reduced signs of innate colitis, thus claiming the importance of ILCreg expansion for the resolution of intestinal inflammation (37). Focusing on the intraepithelial compartment, intraepithelial ILC1s were described to be increased in CD patients (54), which, however, could not be confirmed in a later study (34), making further research on intraepithelial ILCs necessary, especially since their prime location in direct proximity to the intestinal epithelium might predispose them for impacting on the integrity of the epithelial layer. Given that altered ILC frequencies were predominantly observed at inflamed intestinal sites but were absent in non-inflamed areas (34), suggests an active role of intestinal ILCs in inflammatory processes but argues against a primary and disease-predisposing alteration of the ILC compartment in IBD patients.

Based on these numerical ILC alterations observed in the inflamed gut of patients suffering from IBD, the next section will discuss our current knowledge on the functional role of local ILCs in intestinal inflammation with a particular focus on their capacity to interact and regulate IEC functions.

## ILC–IEC Interactions

Enterocytes represent the most frequent cell type in the intestinal epithelium and as such build the fundament for a tight barrier between gut lumen and tissue. This is achieved by tight junctions, which connect the enterocytes to form a robust but selectively permeable wall, allowing a targeted paracellular transport. Together with the transcellular transport through enterocytes, this enables them to absorb nutrients and antigens from the gut lumen in a highly controlled fashion (55), while simultaneously forming an effective first line of defense for pathogens. When disrupted, however, overwhelming invasion of pathogens can cause severe inflammatory immune responses within the intestinal mucosa (56), making a tightly controlled regulation inevitable.

**Figure 2:**
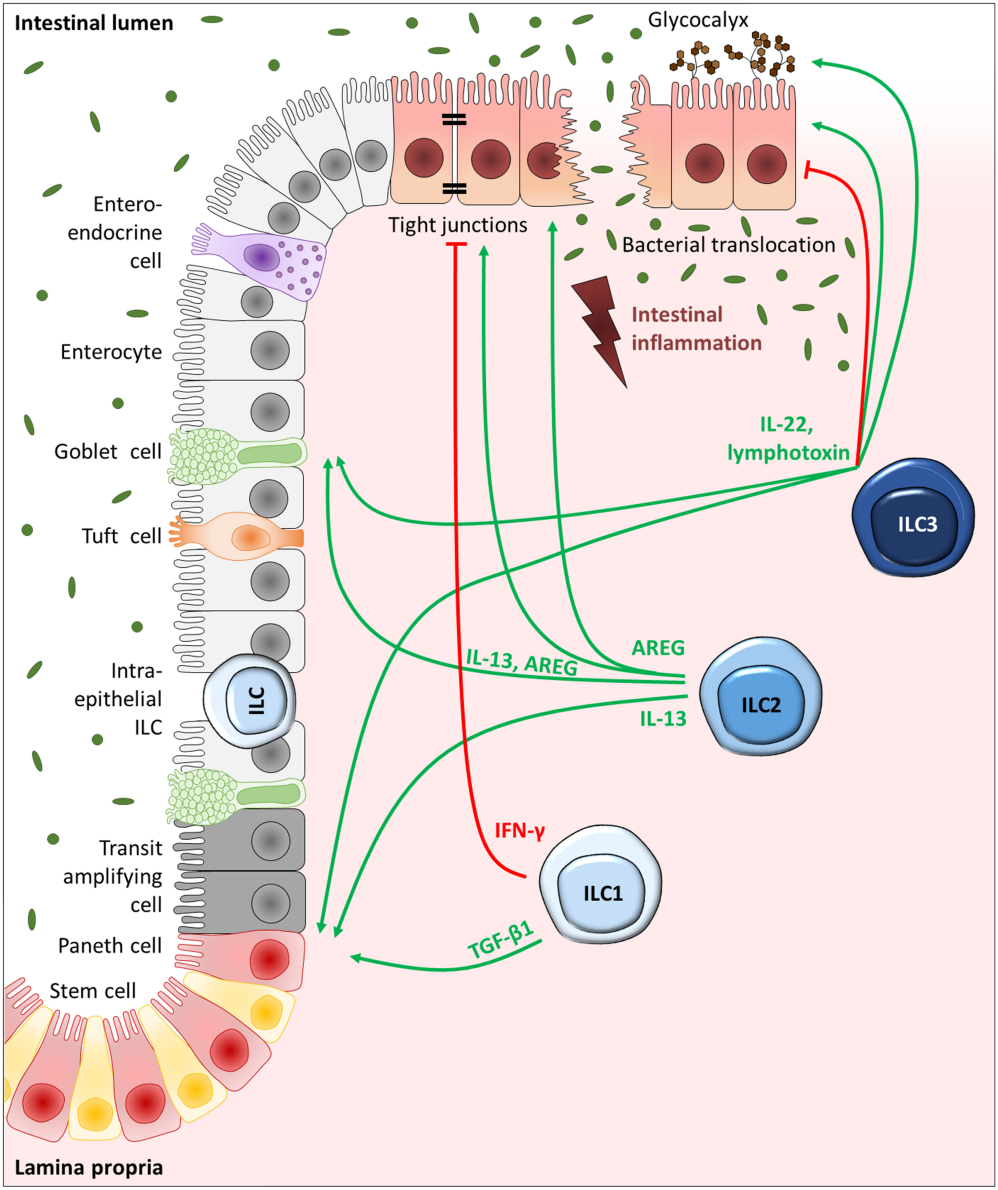
ILC-driven regulation of IECs in intestinal inflammation. Schematic depiction of the intestinal epithelium, consisting of goblet cells, tuft cells, enteroendocrine cells, and M cells dispersed throughout the enterocytes as well as transit-amplifying progenitor cells, paneth cells, and stem cells localized toward the crypt bottom. ILC1s, ILC2s, and ILC3s reside in the mucosa in close proximity to the epithelium or can be directly positioned in between IECs as intraepithelial ILCs, giving them prime positions to interact with IECs. While IECs are important activators of ILCs via the release of selective alarmins, ILCs can in return control the different IEC subtypes via the release of effector cytokines. With the secretion of IL-22 and lymphotoxin, ILC3s can drive stem and progenitor cell proliferation and differentiation. ILC3s can additionally drive mucus production by goblet cells and promote fucosylation of enterocytes. The effect of ILC3-derived IL-22, however, is largely dependent on the microenvironment. ILC2-driven IEC regulation is mainly based on their ability to release IL-13 and AREG, which can trigger stem and progenitor cells, goblet cells as well as robust tight junctions interconnecting enterocytes. Moreover, ILC1-derived TGF-β1, although not a classical type-1 cytokine, can drive stem cell proliferation and differentiation, while IFN-γ secreting ILC1s can weaken the epithelial stability.

Several studies have described a crucial involvement of ILC3s in the regulation of epithelial integrity in the gut, which has been attributed mainly to their capacity to secrete the effector cytokine IL-22 (57–60). Based on the well-known protective functions of IL-22 in IBD (61) and the fact that ILC3s are thought to be the main producers of IL-22 in the homeostatic and inflamed murine gut (58, 60), the favorable functions described for IL-22 on IECs might be largely assigned to ILC3s. Indeed, ILC3-derived IL-22 could be demonstrated to play a protective role on dextran sulfate sodium (DSS)-induced tissue disruption (57–60). DSS-induced colitis represents a widely used model for UC, that is initiated by the chemical disruption of the intestinal epithelium, resulting in colonic inflammation (62), and thereby makes up an ideal model system to study the influence of ILCs on gut barrier integrity. Whereas, under homeostatic conditions ILC3 activity is largely repressed by IL-25 and adaptive immune responses, this repression is downregulated upon DSS-induced tissue disruption and inflammation, allowing the secretion of the protective cytokine IL-22 by intestinal ILC3s (60). In IBD patients an increased IL-22 expression was observed exclusively in ILC3s rather than T cells (63), fortifying the relevance of ILC3-derived IL-22 on gut barrier integrity in humans (Figure 2). Interestingly, the murine norovirus has been shown to take advantage of this ILC3-IL22-IEC-axis in order to protect IECs against tissue disruption (64). Besides the beneficial effect of ILC3s on the barrier function of the intestinal epithelium, they were additionally described to directly foster the glycosylation of eptihelial cells via their effector cytokines IL-22 and lymphotoxin (32) (Figure 2). In general, the colonic glycocalyx functions as additional barrier for pathogens, but also enables communication and adherence of specific bacteria (65). In mice, ILC3s were detected to mediate epithelial fucosylation via the induction of the responsible enzyme fucosyltransferase 2, which turned out to be important for host protection against *Salmonella typhimurium* infection (32).

In IBD patients, decreased frequencies of NKp44^+^ ILC3s were detected at inflamed intestinal sites compared to samples from non-inflamed IBD and non-IBD subjects (17, 34) which was significantly associated with an increased endoscopic disease severity score in both CD and UC patients (34). Since NKp44^+^ ILC3s represent the main producers of IL-22 in the adult intestine (66), the lack of the protective IL-22 effect on the epithelial barrier in IBD patients might at least partially explain the gut barrier disruption. However, in another study, increased IL-22 expression levels have been observed in colonic tissue samples derived from CD and UC patients which could be shown to result from NKp44^+^ ILC3s (63). Interestingly, the presence of fecal microbiota clearly triggered IL-22 production in human LPMCs (63) and potential differences in the composition of the gut microbiota might thus explain, at least partly, the controversy of published data on the intestinal IL-22 levels in IBD patients. Based on findings acquired in innate experimental colitis models (e.g., a model of anti-CD40-induced colitis), ILC3-derived IL-22 could be demonstrated to even have a pathogenic effect (67), indicating a double-edged role of ILC3-derived IL-22 in IBD (Figure 2) that might depend on the local micromilieu and microbiota.

Although less abundant in the gut compared to ILC3s (34), ILC2s can significantly contribute to the preservation and restoration of the intestinal integrity as well (Figure 2). Findings in other organs with a barrier function, like the lung and skin, support this idea. ILC2s could for example be shown to facilitate wound healing of the skin (68), and ILC2-derived amphiregulin (AREG) was suggested as effective player in the epithelial restoration of influenza virus-infected lungs (30). Indeed, first data acquired in the gut could underpin this, showing a tissue-protective role of ILC2s in a mouse model of acute DSS colitis via the secretion of the epidermal growth factor AREG (69). Here the authors suggest a circuit of damaged epithelial cells, releasing the alarmin IL-33 and thereby activating AREG production in ILC2s, which ameliorates DSS-induced tissue disruption, likely via the upregulation of Claudin-1 and Mucin 2 (Muc2) (see section Goblet cells—ILCs for further details on *Muc2*). Claudin-1 represents a well-known tight junction protein (70), whose increased expression favors tightly connected enterocytes forming an efficient barrier in the gut. The observation of a protective function of ILC2-derived AREG was further confirmed in a mouse model of acute gastrointestinal graft-vs.-host disease, in which the intravenous injection of ILC2s could significantly reduce intestinal leakiness in an AREG-dependent manner (71). In contrast to these results, one research group observed detrimental effects of both murine and human ILC2s on the tight junctions of bronchial epithelial cells and epidermal keratinocytes via the secretion of IL-13 (72, 73). These opposing results might indicate the presence of polyfunctional ILC2s (69) which can adapt to local requirement based on the specific microenvironment to gain either protective or destructive functions on epithelial barriers. In the inflamed gut, however, current knowledge indicates a beneficial effect of ILC2s on the intestinal barrier integrity (69, 71). This was additionally indirectly supported by the finding, that the increased intestinal permeability observed in *Itk*^−/−^ mice compared to wildtype mice upon acute DSS colitis was associated with a gut-specific reduction of ILC2s. Injection of IL-2 complexes, however, could both, restore gut ILC2 frequencies and diminish disease severity (74). In IBD patients an increased ILC2 frequency was observed at inflamed intestinal sites (34), suggesting a certain clinical relevance. Just recently, a regulatory subset of IL-10 producing ILC2s was described to have a protective effect in the context of grass-pollen allergy and lung inflammation (75, 76). This was not only mediated by their ability to dampen disease-driving type-2 immune responses (75, 76), but additionally relied on the restoration of the epithelial barrier (75). Whether this regulatory ILC subset is relevant in the inflamed gut as well, has to be determined in future studies, though. First hints, however, imply the potential formation of IL-10 producing ILCs in the inflamed gut mucosa: The transdifferentiation of ILC2s into IL-10^+^ ILC2s or ILCregs interestingly turned out to depend on the vitamin A metabolite retinoic acid (75, 77), elevated levels of which have been reported in patients with active UC (78).

Likewise, ILC1s turned out to accumulate in the inflamed ileum of CD patients (17), pointing toward a functional role in IBD-associated tissue disruption as well. In line with this, the frequency of intraepithelial ILC1s was shown to be increased in the small intestine of CD patients (54). Based on their prime position in immediate proximity to IECs, it can be assumed that intraepithelial ILC1s might play a special role in the regulation of the intestinal epithelial barrier. Further evidence derived from a mouse model of experimental colitis, showing a marked IFN-γ production by intraepithelial ILC1s and to a lesser extent also by plastic ILC3s. Deletion of intraepithelial ILC1s with an anti-NK1.1 antibody was associated with reduced epithelial disruption and a diminished accumulation of inflammatory cells (54), proposing a negative influence of ILC1s on colitis-associated barrier destruction. In the context of celiac disease, increased percentages of intraepithelial NKp44^−^ cytotoxic ILC1s have been reported in the human small intestine, which significantly correlated with an increased IFN-γ production as well as an increased disease severity and epithelial breakdown (79). In addition, enhanced levels of ILC1- and ILC3-derived IFN-γ and TNF-α upon simian immunodeficiency virus infection in rhesus macaques associated with an increased loss of colonic tight junctions, resulting in epithelial instability and increased microbial translocation (80), supporting a detrimental functional role of ILC1s on the maintenance of an intact epithelial gut barrier (Figure 2). A clear proof of ILC1s directly interacting with IECs, however, is still lacking to date, making further research interesting.

Next to enterocytes, the intestinal epithelium consists of highly specialized cell types, including goblet cells, tuft cells, stem cells, paneth cells, enteroendocrine cells, microfold cells, and cup cells (81, 82). While each cell type contributes to the integrity of the epithelium by its unique function, the following section takes a closer look on the direct impact of ILCs on the differentiation and functions of these cell types.

### Goblet Cells—ILCs

Goblet cells represent the secretory cell type of the intestinal epithelium specialized for the production and secretion of mucus components. By the release of mucus, goblet cells form a protective layer over the gut epithelium and thereby relevantly contribute to the intestinal barrier function and the maintenance of mucosal homeostasis (83).

Most prominently, ILC2s could be shown to regulate mucin responses by goblet cells and to thereby protect mice from colonic inflammation. First evidence came from a study by Monticelli and colleagues: Using an acute model of DSS-induced colitis, they could show AREG-derived from IL-33-activated ILC2s to be sufficient for the induction of goblet cell hyperplasia and expression of the mucin *Muc2*. Since this goblet cell activation was accompanied by a decreased overall disease severity of DSS-treated mice (69), this indicates that the protective effect of ILC2s on experimental colitis could be partially mediated by AREG-induced mucus production by goblet cells. This might additionally be supported by ILC2-derived IL-13, which was shown to promote goblet cell differentiation. Applying a coculture system of mesenteric lymph node cells and enteroids, representing a stem cell-derived 3D model system of the small intestinal epithelium, a direct effect of IL-33-induced IL-13 on goblet cell differentiation has been indicated by increased *Muc2* and *Atoh1* expression levels. In this setting, ILC2s were identified as primary source of IL-13 upon IL-33 induction (84), pointing toward a combined action of the ILC2 effector cytokines AREG and IL-13 on goblet cell activation and thus epithelium protection (Figure 2). While a direct effect of IL-13 on goblet cells was demonstrated *in vitro*, no direct impact of AREG could be detected (84), indicating ILC2s to activate goblet cells via both direct and indirect mechanisms. Regarding reported variations in the strength and quality of the impact of ILC2-derived IL-13 and AREG on goblet cell activation (85), it might be important to take into account the potential influence of the local micromilieu, mirrored in different experimental model systems. Another study even expanded these local findings on distal mucosal sites (84). In the context of experimental helminth infection of the gut, it was demonstrated that ILC2s activated by gastrointestinal infection with the helminth *Trichinella spiralis*, do not only activate local goblet cell hyperplasia (86), but could also induce expression of the mucin *Muc5* in the uninfected lung. This phenomenon has been discussed as potential priming mechanism to protect distal sites from secondary helminth infections (84) and strongly implied a systemic relevance of ILC2-triggered goblet cell activation.

During intestinal *Listeria monocytogenes* infection, ILC3s appeared as important inducers of goblet cell differentiation and function, enabling efficient bacteria control. Making use of an *in vitro* coculture transwell assay, the necessity of a direct interaction of lymphotoxin-secreting RORγt^+^ cells and lymphotoxin-β receptor (LTβR) expressing IECs for efficient *Muc2* induction was shown, with ILC3s being suggested as lymphotoxin-expressing interaction partner (87). Though the role of direct ILC3-goblet cell interactions have not been described in IBD yet, a prominent function of IL-22 has been confirmed in the inflamed gut (Figure 2). Using a mouse model of Th2-mediated colitis, IL-22 gene delivery could induce expression of mucus components and goblet cells in a STAT3-dependent manner, which resulted in increased mucus production and thus less severe colitis (88).

In case of *Salmonella typhimurium* infection, IFN-γ-production by an intermediate ILC subtype characterized by NKp46, T-bet, and RORγt expression was claimed to be critical for successful mucus secretion by goblet cells (89). Whether this interaction might play a role in IBD as well, has to be clarified in future studies.

Collectively, ILC2- and ILC3-derived effector cytokines as well as the type-1 cytokine IFN-γ have been shown to strongly impact on goblet cell-driven mucus production and secretion. This allows ILCs to directly regulate the production and composition of the protective mucus layer and to thereby effectively shield IECs from intestinal pathogens.

### Stem and Progenitor Cells—ILCs

The impressive capacity of the intestinal epithelium to renew itself every 3–7 days is driven by a rare cell population positioned at the crypt bottom of the ileum and colon: the intestinal stem cells. Intestinal stem cells can give rise to enterocytes, goblet cells, enteroendocrine cells, and tuft cells throughout life, while simultaneously renewing themselves. Therefore, dividing stem cells partially differentiate into highly proliferative transit-amplifying cells, which successively form terminally differentiated IEC types upon upward migration (90). With their ability to interact with and orchestrate intestinal stem and progenitor cells, ILCs are able to greatly influence overall epithelial functionality despite their rareness, once again demonstrating the direct link between innate immune responses and epithelial restoration.

Most prominently, a positive role of the ILC3-associated cytokine IL-22 on intestinal stem cells has been repeatedly shown (31, 91–95). While a regulatory role of ILC3s on the intestinal epithelium has been proposed shortly after their identification as unique cell population (60), their influence on intestinal stem cells was described only 1 year later. In the context of allogeneic hematopoietic transplantation, Hanash and colleagues identified IL-22 derived from radio-resistant host ILCs in the gut as critical protective factor against epithelial damage during graft-vs.-host disease. With the development of graft-vs.-host reactions, IL-22-secreting ILC frequencies markedly decreased, which was associated with a reduction of IL-22R-expressing intestinal stem cells. Indeed, *Il-22*^−/−^ transplant recipient mice showed an increased loss of intestinal stem cells compared to wildtype mice, which was accompanied by severe disruption of the epithelial barrier integrity. Based on these findings, the authors suggested intestinal ILCs to maintain stem and progenitor cells during tissue damage via the secretion of IL-22, while Paneth cells were proposed to be responsible for basal stem cell maintenance (91). Using an *ex vivo* organoid culture system, they could later translate the importance of IL-22 on stem cell-driven epithelial regeneration to the human system. Moreover, they deciphered the underlying mechanism, showing the dependency of IL-22-mediated intestinal Lgr5^+^ stem cell preservation and resulting organoid growth on STAT3 signaling, while the classical signaling pathways involved in intestinal stem cell maintenance, including Wnt/β-catenin and Notch signaling, were not affected by IL-22. A gene set enrichment analysis of the intestinal stem cell gene signature in STAT3-deficient and -sufficient mice with DSS colitis, reinforced these results under *in vivo* tissue-destructive conditions. Furthermore, the capacity of IL-22 to induce epithelial regeneration appeared to be restricted to intestinal stem and progenitor cells, since the inability of IL-22 to affect Paneth cells could be demonstrated in organoid culture experiments (92). A far-reaching importance of IL-22-secreting ILC3s on stem cell-driven intestinal barrier maintenance by the interaction with crypt stem cells could be further established in a setting of chemotherapy-induced small intestinal tissue disruption (31). Beyond that, ILC3-derived IL-22 turned out to contribute to the initiation of the DNA damage response in intestinal stem cells, preventing them from the acquisition of potential mutations that might cause intestinal cancer development. This was mediated via aryl hydrocarbon receptor (AhR) signaling in ILC3s as well as γδ T cells in response to genotoxic stress triggered by dietary compounds (93). A recent study differentiated more explicitly between the maintenance and proliferation of intestinal stem cells, showing that upon acute damage of the small intestine by methotrexate, ILC3-derived IL-22 was primarily relevant in protecting and preserving stem cells in a STAT3-dependent manner, while ILC3-driven, but IL-22-independent amplification of the Hippo-Yap1 pathway in stem cells turned out to mediate crypt cell proliferation in a SFK-dependent fashion. The authors thus proposed that ILC3s might not only interact with Lgr5^+^ stem cells for their maintenance, but might also directly or indirectly trigger proliferation of damage-linked progenitor cells to restore an intact epithelial barrier after tissue disruption (94). The exact mechanisms through which ILC3s drive epithelial restoration independently of IL-22, however, still need to be evaluated. Somehow in contrast, another study proposed a role of high IL-22 concentrations primarily for the amplification of transit-amplifying progenitor cells rather than intestinal stem cells based on findings in an *in vitro* ileal organoid culture model. There the authors observed a negative effect of 500 pmol/l IL-22 on organoid survival, whereas remaining organoids showed an increase in size, which was suggested to results from highly proliferating transit-amplifying progenitor cells. The principle idea for analyzing IL-22 concentrations as high as 500 pmol/l was derived from a computational modeling of the local ILC3-secreted IL-22 concentration in the stem cell niche (95). To date, however, absolute IL-22 concentrations in the microenvironment of intestinal stem cells have not been experimentally confirmed yet. Thus, more research is necessary to determine the functional role of ILC3-derived IL-22 on distinct stem and progenitor cell subsets under defined inflammatory conditions. Taken together, multiple studies were able to identify ILC3s as key players in preserving and rebuilding an intact epithelial barrier in the gut after tissue damage (Figure 2). This can be mediated by a direct interaction of IL-22-secreting ILC3s and IL-22R-expressing intestinal stem cells and by ILC3-driven activation of Yap1 signaling in stem cells. ILC3s are obviously able to rapidly rebuild an efficient gut barrier upon various kinds of tissue disruptions and, in addition, can prevent intestinal cancer development originating from DNA damage in stem cells.

Besides a fundamental role of ILC3s on intestinal stem and progenitor cells, ILC2s were suggested to interact with intestinal progenitor cells as well. In helminth infection models with *Nippostrongylus brasiliensis* and *Heligmosomoides polygyrus*, they have been demonstrated to induce goblet and tuft cell differentiation via the secretion of IL-13 (96). In general, the latter represent a chemosensory IEC subset with striking similarities to our taste buds. Thus, tuft cells are suggested to “taste” luminal signals unable to cross the intestinal barrier to trigger a specific response in the intestinal tissue (97), though their exact function has long been undetermined and is still insufficiently clarified. However, a pivotal role of intestinal tuft cells has been suggested during helminth infections, during which their rare number literally explodes (96, 98). Since tuft cells turned out to be the major source of IL-25 in the intestinal epithelium, the functional relevance of ILC2-driven tuft cell hyperplasia upon helminth infection was suggested to lie in the activation of ILC2s themselves via the secretion of IL-25, in order to mount an efficient anti-helminth immune response. Indeed, in systemic and epithelium-specific *Il-25* knockout mice helminth infections were only inefficiently cleared (96, 98). Similarly, *Trpm5*^−/−^ mice, which are unable to transduce taste signals in tuft cells, are characterized by an increased worm burden after helminth infection compared to wildtype mice. This could be restored upon intraperitoneal injection of IL-25 (98). Thus, a regulatory circuit was suggested with IL-25-secreting tuft cells stimulating IL-13 release by local ILC2s, which in turn triggers tuft cell proliferation from progenitor cells via a positive feedback mechanism, finally resulting in successful worm clearance (96). Next to this pathologic context, this circuit was suggested to be important even under homeostatic conditions, showing in uninfected *Il-25*^−/−^ and *Il-4ra*^−/−^ mice that constitutive IL-25 secretion by tuft cells as well as ILC2-derived IL-13 were important to maintain intestinal tuft cell numbers in naive mice (96). Collectively, intestinal tuft cells can be positively regulated by ILC2-derived IL-13 (Figure 2), leading to tuft cell expansion via activating crypt progenitor cells and thus directly regulating anti-helminth responses, raising the question whether this tuft cell-ILC2 circuit might play a role in IBD as well. Indeed, decreased tuft cell counts were recently described in CD patients at inflamed ileal tissue sites (99). Moreover, using a model of acute DSS colitis, a beneficial role of *Dclk*^+^ tuft cells on intestinal barrier integrity was observed (100). In line with this, reduced IL-25 levels were found in the inflamed gut mucosa of IBD patients with active disease, which correlated with an increased disease severity (101), suggesting the loss of intestinal tuft cells and their IL-25 secretion as disease-driving factor in IBD. In the murine ileum, tuft cells could be reconstituted together with local ILC2 frequencies and classical type-2 cytokines by the administration of the microbiota-derived metabolite succinate, finally resolving ileal inflammation (99). This implies an *in vivo* relevance of the ILC2-driven tuft cell regulation upon intestinal inflammation in CD patients and might additionally explain the recently described protective role of helminth infections in the development of IBD (99).

Also for ILC1s a regulatory role on intestinal stem cells has been described lately (Figure 2). Though not representing a classical ILC1-associated cytokine, ILC1-derived TGF-β1 was able to specifically induce the expression of the variant 6 of the stem cell marker CD44 (*Cd44v6*) in the intestinal epithelium. This resulted in enhanced crypt budding of small intestinal organoids *in vitro* via p38γ-induced proliferation. Having observed an enhanced expression of *CD44v6* in enlarged intestinal crypts in the inflamed tissue of IBD patients as well (45), a positive regulatory role of ILC1s on epithelial expansion upon inflammation might be of significance *in vivo*.

Collectively, ILC1s, ILC2s, and ILC3s are described to have a beneficial impact on epithelial restoration and growth upon inflammation, resulting from direct interactions with intestinal crypt stem and progenitor cells.

### Further IEC Subtypes—ILCs

Next to enterocytes, goblet, progenitor and stem cells as well as tuft cells, the IEC fraction additionally consists of enteroendocrine, paneth, and M cells, each of these subtypes contributing to the diverse functions of the intestinal epithelium. Enteroendocrine and paneth cells for instance are known for the secretion of hormones and antimicrobial peptides, respectively, while M cells can transport antigens from the gut lumen into the intestinal tissue and thus serve the intestinal immune system (81).

To date, no interaction of ILCs with these IEC subtypes has been shown but might be implicated by the presence of potential interaction sites between IECs and ILCs. In case of enterochromaffine cells for example, which constitute a subset of enteroendocrine cells and are critical for the production of serotonin, expression of the IL-13 receptor α1-chain could be demonstrated (102), implicating a potential signal induction in enterochromaffine cells by ILC2-derived IL-13. Enteroendocrine cells might in addition be positively regulated by IL-22 released from ILC3s. As suggested in a mouse model of *Citrobacer rodentium* infection, secretion of antimicrobial peptides, primarily of the Reg family, from epithelial cells turned out to depend on IL-22 production from IL-23 responsive cells of the innate immune system (103), providing a direct link between ILC3s and the bacterial defense mechanisms of the intestinal epithelium. Moreover, the localization of paneth cells at the crypt bottom exposes them to an environment that was suggested by computational modeling to be characterized by particularly high ILC3-derived IL-22 levels (95). Together with the recently shown importance of IL-22 signaling for paneth cell differentiation and effector functions (104), this predisposes ILC3s as potential regulators of paneth cells. Future research will help to clarify the biological relevance of these suggested ILC-IEC interactions in IBD.

## ILC–IEC Interactions as Therapeutic Target

Given the preferential accumulation of ILCs at mucosal surfaces in close proximity to the epithelium (10, 11) as well as their rapid and early activation as part of the innate immune system (12–14), makes them ideal interaction partners of IECs. Moreover, the ILC-driven regulation of IECs is of particular importance as the integrity of the epithelial barrier is critical to preserve a stable and efficient control of bacterial translocation, which might otherwise trigger the initiation of mucosal inflammation. Based on our current knowledge, ILC3s represent the main helper ILC population in the healthy human gut (33, 34) (Figure 1) with an incredible ability to secrete large amounts of the effector cytokine IL-22 (58, 60). Together with the release of lymphotoxin, ILC3-derived IL-22 was ascribed a predominantly protective effect on enterocytes, goblet cells and even crypt stem and progenitor cells (57–60, 87, 91, 92). Therefore, the observed decrease of intestinal ILC3s (17, 34, 44) in the inflamed mucosa of IBD patients might contribute to local inflammatory responses in IBD. However, the effect of ILC3-derived IL-22 on IECs appeared to highly depend on the surrounding micromilieu (67) and thus has to be treated with care. With an increasing number of intestinal ILC1s in CD (17, 44), their TGF-β1-mediated beneficial effect on crypt stem cells (45) might contribute to the resolution of intestinal inflammation by promoting the reconstruction of an intact epithelial barrier. In contrast, via the secretion of IFN-γ, ILC1s were suggested to additionally have a negative influence on tight junctions (54, 79, 80), implying functional subgroups of ILC1s. Despite their overall low number in the healthy and inflamed intestine, ILC2s could be demonstrated to favor mucosal healing and disease control on the level of enterocytes, goblet cells, tuft cells and crypt stem cells by secreting AREG and IL-13 (69, 71, 84). Overall, the described ILC-IEC interactions prove once again, that a rare cell population like ILCs can have an extensive impact on disease induction, progression, and resolution irrespective of their small cell number (Figure 2), making the interference with ILC-driven IEC regulation an interesting new therapeutic option. Targeting ILCs appears to be particularly elegant regarding the ability of intestinal ILCs to regulate both, epithelial integrity as well as mucosal immune responses. However, this also makes clear that the development of ILC-modulating therapeutic strategies will require a careful fine-tuning to prevent accidental ILC-mediated immune cell activation upon boosting protective ILC-IEC interactions. Notably, no fully ILC-specific target structures exist these days, which is mainly due to the shared key features of ILCs and Th cells. This makes it currently impossible to clearly differentiate between ILC- and T cell-targeting therapeutic approaches. A broad overview of ILC-related therapeutic strategies was given by Goldberg and colleagues, postulating the following points of potential attack: targeting ILC effector functions by therapeutically modulating their activity, intracellular signaling, and effector cytokine production or targeting local ILC numbers by interfering with their survival and trafficking (105). To date, most of the postulated therapies involving ILC targeting aim at the inhibition of pro-inflammatory ILC functions. However, these strategies need to be critically revised, since inhibition of total ILC functions might not only favor resolution of inflammation but might additionally abolish their mainly positive influence on the intestinal epithelium upon inflammation. Thus, novel therapeutic approaches might focus on the controlled stimulation of specific and defined aspects of ILC functions, rather than mediating a broad or complete blockade of ILC activation. The following section will therefore focus on potential therapeutic options, which might allow to directly and specifically interfere with defined ILC-IEC interactions.

Given the great ability of ILC3-derived IL-22 to preserve the intestinal epithelium (57–60) and the fact that the IL-22 receptor (IL-22R) is absent on hematopoietic cells (106), ILC3-derived IL-22 appears as an attractive therapeutic target, since this might enable to support the protective effect of IL-22 on epithelial cells without triggering pro-inflammatory immune responses in parallel. Accordingly, different strategies to boost IL-22-mediated epithelial regeneration and preservation have been discussed. On the one hand, IL-22 can be supplemented directly and, on the other hand, therapeutic activation of endogenous IL-22 production and signaling can be targeted. In the latter category, a site-specific *Il-22* gene delivery system was suggested and proven to improve signs of intestinal inflammation in a preclinical mouse model of Th2-driven colitis (88). However, there is still plenty of safety issues to be addressed carefully until this local gene-delivery strategy might be translated to the human system. Alternatively, therapeutic activation of the IL-22 pathway might represent an interesting strategy. To date, most promising approaches involve AhR-mediated IL-22 induction, which is of functional relevance in ILC3s as well (107, 108). Activation of the AhR pathway can be achieved, for instance, by dietary compounds (107) or microbiota-driven metabolization of tryptophan to the AhR ligand indole-3-aldehyde (109, 110), the latter of which was demonstrated to augment intestinal inflammation in murine models (109, 110). Similarly, indigo naturalis, which has been traditionally used in Chinese medicine, turned out to mediate its protective effect via AhR-mediated IL-22 production in ILC3s as well, resulting in reduced disease severity in several murine models of colitis (111). Moreover, especially a short-term treatment with indigo naturalis has been identified as effective treatment in patients with UC (112), whereas 8 weeks of administration cannot yet be fully excluded to potentially be associated with severe adverse events (113). Importantly, reduced AhR activation was observed in fecal samples from IBD compared to healthy subjects (110). Therefore, together with other AhR ligand candidates, including FICZ (114) and ABX464 (115), tryptophan and indigo naturalis might be promising therapeutic strategies in IBD patients by activating endogenous IL-22 production from intestinal ILC3s in order to protect and restore the intestinal epithelium. Since, however, AhR activation can trigger multiple signaling pathways dependent on its ligand (116), potential therapeutic interference with AhR signaling would have to be carefully controlled and monitored. Another strategy to target IL-22 signaling might involve blocking of the soluble IL-22 receptor IL-22 binding protein (IL-22BP), which functions as endogenous inhibitor of IL-22 by capturing IL-22 with high affinity and thus suppressing IL-22 signaling through its membrane-bound receptor (117). A beneficial effect of blocking IL-22BP in intestinal inflammation has been implicated by experimental colitis models, showing a detrimental role of local IL-22BP. Local gene delivery of *IL-22bp*, for instance, hindered IL-22-mediated goblet cell proliferation in a mouse model of acute DSS colitis (88). Moreover, CD4^+^ T cell-derived IL-22BP could be shown as driver of intestinal inflammation in the model of adaptive transfer colitis using *Il-22bp*-sufficient and -deficient donor T cells (118). The other way around, IL-22bp-deficient rats recovered significantly faster from first signs of colitis upon DSS treatment compared to wildtype control animals, which could be traced back to the protective effects of efficient IL-22 signaling on the epithelial barrier (119). Since inflamed areas in IBD patients are characterized by an increased expression of *IL-22BP* (118, 119), blocking IL-22BP might potentially have therapeutic potential in humans as well. Alternatively, recombinant human IL-22 fusion proteins, like F-652 or UTTR1147A, allow direct IL-22 supplementation with the advantage of increased IL-22 stability and thus extension of the IL-22-induced beneficial effects. In case of UTTR1147A, efficient STAT3 activation via the IL-22 receptor could be demonstrated *in vitro*, resulting in protective *in vivo* effects in a murine colitis model (120). Moreover, the therapeutic efficacy of F-652 has been shown in a mouse model of graft-vs.-host disease (92) and its safety profile has even been validated successfully in a clinical trial (121), paving the way for further clinical development. Irrespective of the afore mentioned strategies, fine-balancing of any therapeutic interference with ILC3-driven IL-22 secretion and individual patient selection will be of critical importance, since next to its protective effects on IECs, a detrimental potential of IL-22 on the epithelial barrier has been observed in selected mouse models, as for instance in the anti-CD40-induced innate colitis model (67). Likewise, uncontrolled IL-22 was suggested to favor formation of colitis-associated colon cancer (122), allowing IL-22 modulations only in a highly controlled fashion.

Boosting the ILC2-progenitor cell-tuft cell circuit might represent another innovative, yet currently still hypothetical therapeutic option in IBD patients. Indeed, data from a murine study indicated that succinate supplementation resulted in the amelioration of intestinal disease, which was attributed to an activation of the tuft cell-ILC2 circuit (99). Even though expansion of the helminth-sensing tuft cells by defined microbiota-derived metabolites has not directly been targeted in IBD patients yet, helminth infections themselves have been discussed to prevent intestinal inflammation, though to date with controversial outcomes (123–126). Thus, it appears promising to pursue strategies that aim on a more specific therapeutic activation of the tuft cell-ILC2 circuit by specific microbial metabolites, like succinate.

Hypothetically, a direct transfer of *ex vivo* expanded and specifically activated human ILCs might represent another future therapeutic strategy, aiming at the restoration of a functional epithelial barrier. Motivated by the first promising results achieved by the adoptive transfer of regulatory T cells (127, 128) with a phase I clinical trial currently running in UC patients (NCT04691232) (129), the transfer of other protective immune cell populations might also be of advantage for IBD patients. While there already exist established protocols for the *ex vivo* expansion and differentiation of human ILCs (48, 130, 131), a targeted *ex vivo* generation of specific ILC subsets with distinct effector functions is certainly still an unsolved challenge that needs to be addressed as a first step on the way to a potential clinical translation. However, pursuing this idea, the therapeutic use of *ex vivo* expanded primarily AREG-producing ILC2s might, for instance, be desirable, based on their beneficial effect observed in a mouse model of intestinal inflammation (69). Nevertheless, many more questions need to be answered, until adoptive ILC transfer might be further discussed as therapeutic strategy in IBD. For example, it will be important to define the *in vivo* stability of the intended ILC phenotype, the capacity of transferred ILCs to accumulate at the site of epithelial tissue damage as well as their potential risk to drive inflammation rather than epithelial healing under *in vivo* conditions.

In summary, the here described insights into the capacity of helper ILCs to impact on the fate of IECs clearly point to these innate mucosal immune cells as key regulators of the gut barrier integrity and as an interesting and still largely unexplored research topic with great therapeutic potential in the clinical context of IBD.

## Author Contributions

All authors listed have made a substantial, direct and intellectual contribution to the work, and approved it for publication.

## Conflict of Interest

MN has served as an advisor for Pentax, Giuliani, MSD, Abbvie, Janssen, Takeda and Boehringer. The remaining authors declare that the research was conducted in the absence of any commercial or financial relationships that could be construed as a potential conflict of interest.
